# Regulation of glycolysis and the Warburg effect in wound healing

**DOI:** 10.1172/jci.insight.138949

**Published:** 2020-09-03

**Authors:** Roohi Vinaik, Dalia Barayan, Christopher Auger, Abdikarim Abdullahi, Marc G. Jeschke

**Affiliations:** 1 Sunnybrook Research Institute, Toronto, Canada.; 2 Department of Surgery, Division of Plastic Surgery, and; 3 Department of Immunology, University of Toronto, Toronto, Canada.; 4 Ross Tilley Burn Centre, Sunnybrook Health Sciences Centre, Toronto, Canada.

**Keywords:** Inflammation, Therapeutics, Glucose metabolism, Skin

## Abstract

One of the most significant adverse postburn responses is abnormal scar formation, such as keloids. Despite its prolificacy, the underlying pathophysiology of keloid development is unknown. We recently demonstrated that NLRP3 inflammasome, the master regulator of inflammatory and metabolic responses (e.g., aerobic glycolysis), is essential for physiological wound healing. Therefore, burn patients who develop keloids may exhibit altered immunometabolic responses at the site of injury, which interferes with normal healing and portends keloid development. Here, we confirmed keloid NLRP3 activation (cleaved caspase-1 [*P* < 0.05], IL-1β [*P* < 0.05], IL-18 [*P* < 0.01]) and upregulation in Glut1 (*P* < 0.001) and glycolytic enzymes. Burn skin similarly displayed enhanced glycolysis and Glut1 expression (*P* < 0.01). However, Glut1 was significantly higher in keloid compared with nonkeloid burn patients (>2 SD above mean). Targeting aberrant glucose metabolism with shikonin, a pyruvate kinase M2 inhibitor, dampened NLRP3-mediated inflammation (cleaved caspase-1 [*P* < 0.05], IL-1β [*P* < 0.01]) and improved healing in vivo. In summary, burn skin exhibited evidence of Warburg-like metabolism, similar to keloids. Targeting this altered metabolism could change the trajectory toward normal scarring, indicating the clinical possibility of shikonin for abnormal scar prevention.

## Abstract 2

Burn skin exhibits Warburg-like metabolism, similar to keloids, and targeting altered glucose metabolism could mitigate scarring.

## Introduction

Wound healing is a complex and carefully coordinated physiologic response to a cutaneous injury inflicted in conditions such as surgery, trauma, or burns. Deregulation of this process following insult to the reticular dermis can result in aberrant scar formation, such as keloids (1–3). Despite their common occurrence, keloids remain one of the most challenging conditions to successfully treat and are associated with pruritus, pain, and contractures, often leaving a significant functional and psychosocial impact on patients. They also occur in response to other nonburn trauma including surgical incisions and lacerations, providing a further impetus for delineating pathomechanisms underlying keloid formation.

Evidence suggests that fibroblasts isolated from keloids undergo a metabolic reprogramming from oxidative phosphorylation to aerobic glycolysis, known as the “Warburg effect” in cancer cells (4, 5). Owing to their tumor-like nature, keloid cells have a higher glucose influx coupled with elevated lactate production compared with normal fibroblasts. Interestingly, a similar metabolic profile is seen in nonkeloid hyperproliferative conditions that result in an analogous excessive extracellular matrix (ECM) production to keloids (e.g., scleroderma, surgical/medical/radiation-induced fibrosis) (6). Indeed, these human and murine fibrosis models demonstrate an upregulation in glycolytic enzymes and glucose transporters coupled with increased lactate production, highlighting a similar pathological behavior to keloid and tumor cells (6, 7).

Interestingly, upregulation in glycolysis and glucose uptake is also seen in conditions of physiologic healing and burn injuries, although to a lesser extent (8–12). In the context of burns, the process of cutaneous wound healing is initiated by an inflammatory response at the site of injury generated by nucleotide-binding and oligomerization domain, leucine rich repeat and pyrin domain containing 3 (NLRP3) inflammasome, the master regulator of inflammatory and metabolic responses (13–15). Upon activation, NLRP3 serves as a danger-sensing platform, facilitating cleaved caspase-1 processing and promoting the release of IL-1β and IL-18. While inflammation is considered beneficial for adequate wound closure and repair, it paradoxically has also been linked to increased fibrosis in multiple models of repair (6, 16, 17). Thus, a more detailed understanding of mechanisms controlling the inflammatory response and how inflammation directs the healing process is imperative for clinical management of pathological scarring.

The aim of this study was to delineate the role of altered glucose metabolism and inflammation in normal burn wound repair versus keloid formation. We hypothesized that the Warburg effect would promote chronic NLRP3 inflammasome activation in keloids. Furthermore, we postulated that physiologic postburn wound healing would exhibit a similar metabolic shift toward aerobic glycolysis and lactate production. However, aberrant glucose metabolism at the site of injury could promote a chronic inflammatory state, predisposing certain patients to keloid formation. Therefore, we propose that early identification of patients with increased scarring risk is possible by profiling based on local inflammatory and glycolytic responses. These patients can subsequently be treated with agents that target aberrant inflammation and glycolysis, minimizing their risk of future scarring.

## Results

### NLRP3 inflammasome is activated in human keloid tissue.

**Figure 1:**
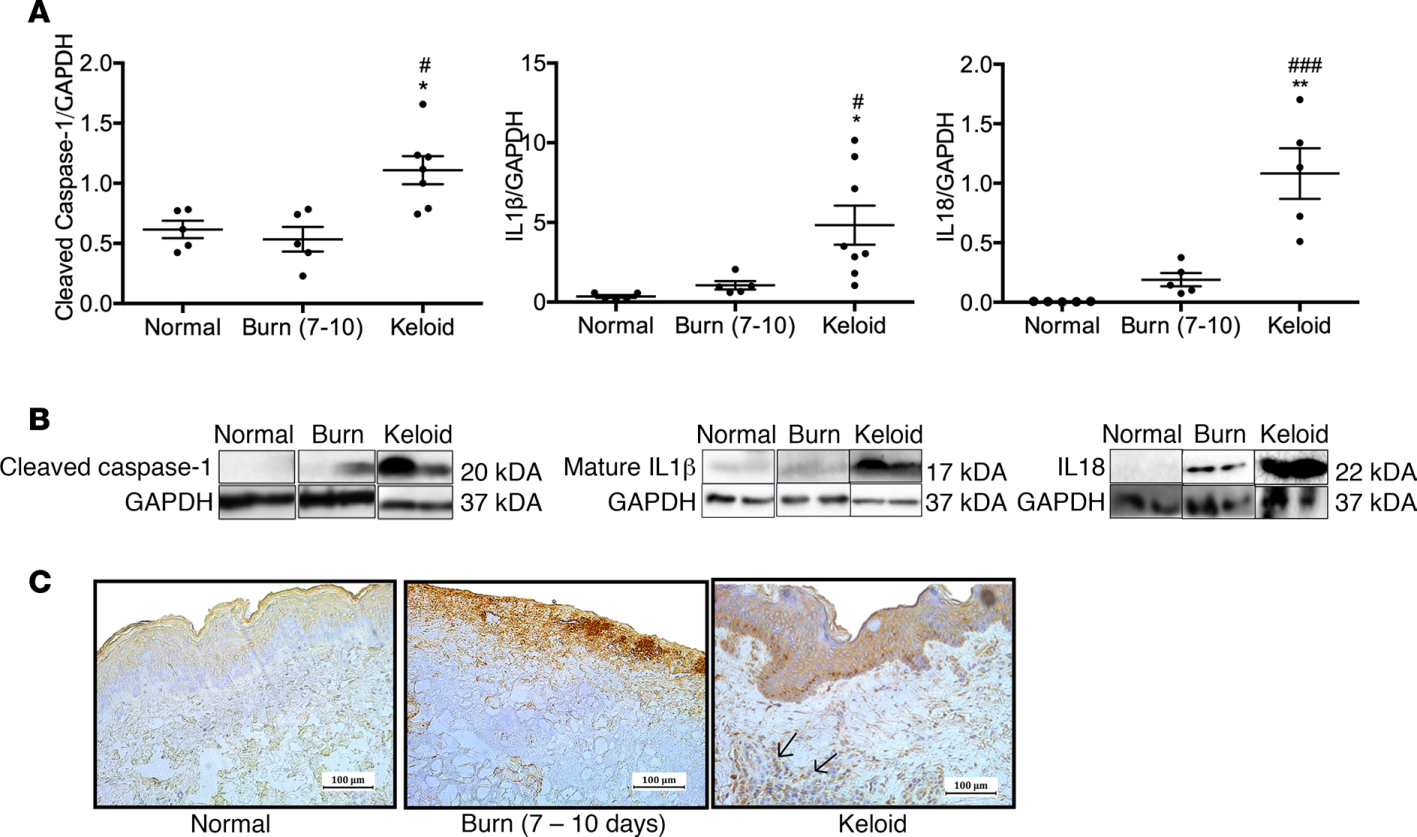
Activation of NLRP3-mediated inflammation in keloids. (**A**) Protein expression of cleaved caspase-1 (left) (*n* = 5 normal skin, *n* = 5 burn skin, *n* = 8 keloid), mature IL-1β (center) (*n* = 5 normal skin, *n* = 5 burn skin, *n* = 8 keloid), and IL-18 (right) (*n* = 5 normal skin, *n* = 5 burn skin, *n* = 5 keloid). (**B**) Representative cropped Western blots for cleaved caspase-1 (left), mature IL-1β (center), and IL-18 (right). (**C**) Immunohistochemical staining for NLRP3 in normal skin, burn skin (7–10 days), and keloid tissue indicates NLRP3^+^ cells in keloid dermis (arrows). Values are presented as mean ± SEM. Experiments were conducted twice. One-way ANOVA; *P < 0.05 and **P < 0.01 keloid versus burn; ^#^*P* < 0.05 and ^###^*P* < 0.001 keloid versus normal.

Activation of NLRP3 inflammasome after burns occurs acutely in human skin (0–2 days after burn) and returns to baseline by 7–10 days (15). We determined if NLRP3-mediated inflammation is still activated beyond this time point in keloids by measuring protein levels of cleaved caspase-1 and IL-1β in keloids compared with burn skin (7–10 days after burn, average age 53 years and total body surface area [TBSA] 39%) and normal skin (Figure 1, A and B). Keloids demonstrated elevated cleaved caspase-1 compared with burn and normal skin (1.43 vs. 0.53, *P* < 0.05; 1.43 vs. 0.61, *P* < 0.05), elevated mature IL-1β compared with burn and normal skin (4.83 vs. 1.06, *P* < 0.05; 4.83 vs. 0.36, *P* < 0.05), and elevated IL-18 compared with burn and normal skin (1.08 vs. 0.19, *P* < 0.01; 1.08 vs. 0.005, *P* < 0.001). Furthermore, immunohistochemical staining for NLRP3 was positive in the keloid dermis, with no clear evidence of NLRP3^+^ cells in normal and burn skin (Figure 1C). Taken together, these results suggest that NLRP3-mediated inflammation is present in keloids and may contribute to a persistent inflammatory state.

Interestingly, recent studies indicated that there is a link between inflammatory and glycolytic responses; namely, NLRP3 priming and activation is regulated by increased glucose influx and glycolysis, and augmented ATP production (18–20). Therefore, altered glucose metabolism at the site of injury could contribute to a chronic inflammatory state, increasing the risk of keloid development. Consequently, we assessed local glucose uptake in burn skin compared with keloids and normal skin.

### Elevated Glut1 in burn patients who develop keloids.

**Figure 2:**
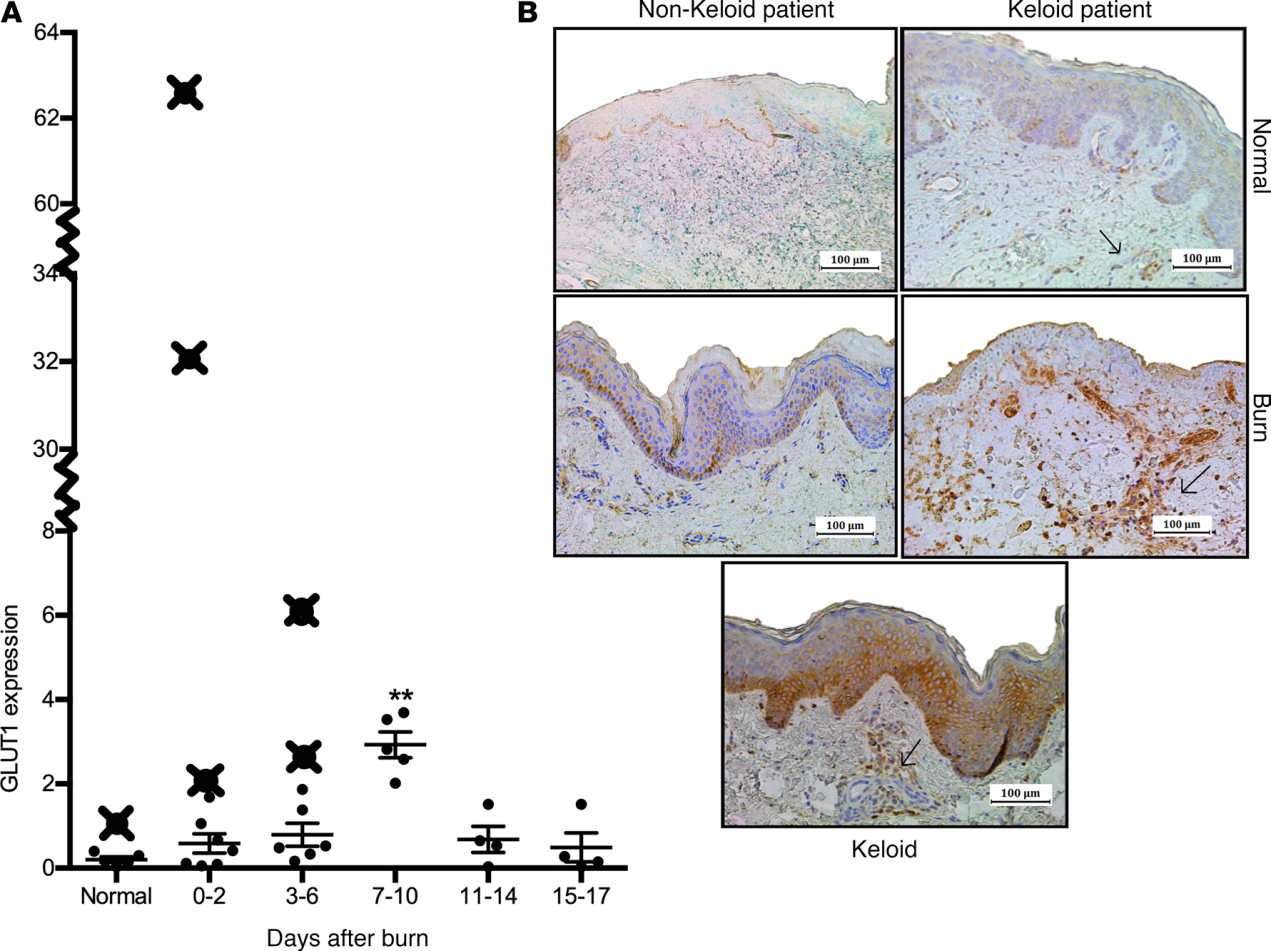
Burn patients who develop keloids have prior indications of altered glucose metabolism. (**A**) *GLUT1* gene expression in burn and normal skin samples obtained from burn patients before the development of keloids indicates higher expression compared with typical values seen in nonkeloid patients (≥ 2 SDs above mean). Similarly, keloids exhibited increased Glut1 staining. (**B**) Immunohistochemical staining for Glut1 in skin from keloid patients indicates more Glut1^+^ cells in basal epidermal and dermal layers compared with nonkeloid counterparts (normal skin, top panel; burn skin 7–10 days after burn, bottom panel). Individual data points (X) denote gene expression values for individual patients. Values are presented as mean ± SEM. Experiments were conducted twice. One-way ANOVA; **P < 0.01 burn versus normal.

We assessed whether burn patients who develop keloids demonstrate evidence of increased glucose uptake compared with nonkeloid burn patients by measuring expression of the facilitative glucose transporter Glut1. Indeed, keloids exhibit Glut1 positivity, indicative of increased local glucose uptake and glycolysis activation (Figure 2A) (6). Therefore, we conducted a time-course analysis of *GLUT1* gene expression in burn skin of nonkeloid patients and showed that *GLUT1* increases significantly at 7–10 days compared with normal skin (3.22 vs. 0.20, *P* < 0.01) (Figure 2A).

Potentially, differences in skin *GLUT1* expression in nonkeloid versus keloid burn patients could serve as an indicator for increased keloid risk. For the next component of our study, we obtained keloid patient skin samples during admission to determine if *GLUT1* gene expression is greater than that of nonkeloid patients at similar time points after burn. Here, we showed that skin samples obtained during admission from keloid burn patients exhibited significantly elevated *GLUT1* expression, defined as more than 2 SDs above the average at the particular time point (Figure 2A). Moreover, staining for Glut1 in normal and burn tissue from keloid patients indicates greater Glut1 positivity in basal epidermal and dermal layers compared with skin obtained from nonkeloid patients (Figure 2B). Given the differences in *GLUT1* expression between keloid tissue, burn, and normal skin, we subsequently assessed expression of other key glycolytic enzymes.

### Glycolysis is upregulated after burn in human skin in an analogous manner to keloids.

**Figure 3:**
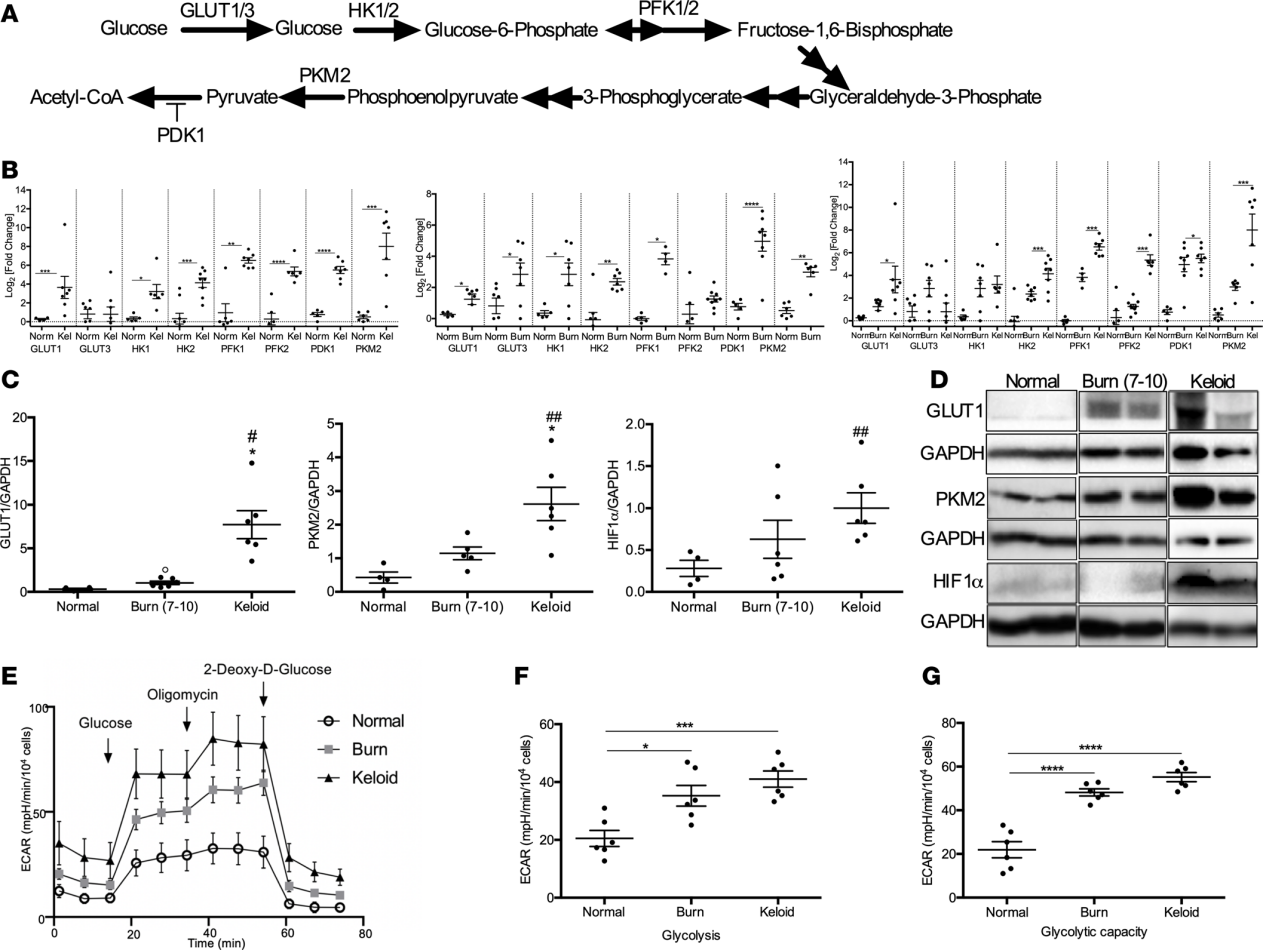
Altered glucose metabolism in keloid and burn tissue compared with normal skin. (**A**) Schematic depicting critical glycolytic enzymes evaluated in keloid and burn tissue. (**B**) Gene expression studies for *GLUT1, GLUT3, HK1, HK2, PFK1, PFK2, PDK1,* and *PKM2* in keloid tissue compared with normal skin (left), burn skin compared with normal skin (center), and all 3 tissues (right) (*n* = 6–8). (**C**) Protein expression of Glut1 (*n* = 4 normal skin, *n* = 6 burn skin, *n* = 6 keloid), PKM2 (*n* = 5 normal skin, *n* = 5 burn skin, *n* = 6 keloid), and Hif1α (*n* = 5 normal skin, *n* = 6 burn skin, *n* = 6 keloid). (**D**) Representative cropped Western blots for Glut1, PKM2, and Hif1α. (**E**) Seahorse XF96 glycolysis stress test performed on fibroblasts from normal skin (*n* = 5), burn (*n* = 8), and keloid (*n* = 6) tissues. (**F** and **G**) Measurements of glycolysis (**F**) and glycolytic capacity (**G**) were made possible using the Seahorse XF stress test reporter generator. Values are expressed as log_2_ (fold change) relative to normal skin, presented as mean ± SEM. Experiments were conducted twice. Student’s *t* test and 1-way ANOVA; *P < 0.05, **P < 0.01, ***P < 0.001, and ****P < 0.0001 keloid versus burn; ^#^*P* < 0.05 and ^##^*P* < 0.01 keloid versus normal; °*P* < 0.05 burn versus normal.

Rapidly proliferating keloid cells — and, by extension, normal cells involved in the proliferative phase of physiologic wound healing — depend on aerobic glycolysis for growth (21). This in turn putatively perpetuates the inflammatory response, increasing the risk for keloid formation. Previous studies have demonstrated concurrent upregulation of Glut1 and several glycolytic genes (e.g., hexokinase 1 [*HK1*] and *HK2*, phosphofructokinase 1 [*PFK1*] and *PFK2*, pyruvate dehydrogenase kinase 1 [*PDK1*], and pyruvate kinase M2 [*PKM2*]) in murine and human skin fibrosis, indicative of enhanced glycolysis (Figure 3A) (22, 23). We showed a similar observation in keloid samples compared with normal skin (Figure 3B, left panel). Keloid tissue demonstrated increased expression of *GLUT1* (2.67 vs. 0.27, *P* < 0.001), *HK1* (3.20 vs. 0.35, *P* < 0.05), *HK2* (4.14 vs. 0.33, *P* < 0.001), *PFK1* (6.51 vs. 0.95, *P* < 0.01), and *PFK2* (5.35 vs. 0.28, *P* < 0.0001). Expression of *PDK1*, which blocks entry of pyruvate into the tricarboxylic acid cycle and promotes lactate production, was also upregulated (5.50 vs. 0.76, *P* < 0.0001) (22, 24). Since *PDK1* and *GLUT1* are PKM2-targeted genes, we subsequently analyzed expression of PKM2 in keloid tissue, which was upregulated relative to normal skin (7.58 vs. 0.29, *P* < 0.001) (Figure 3B). When comparing protein expression for Glut1 and PKM2, we demonstrated similar findings of elevated levels in keloid tissue compared with normal skin (5.84 vs. 0.31, *P* < 0.05 for Glut1; 2.92 vs. 0.63, *P* < 0.01 for PKM2) (Figure 3, C and D). Additionally, Hif1α protein expression in keloids was increased (1.00 vs. 0.28, *P* < 0.001). Hif1α is responsible for upregulating Glut1 and glucose uptake, as well as various glycolytic enzymes, and is activated in hypoxic conditions such as keloids. Although Hif1α levels in burn skin were not on par with keloid tissue, expression appeared to be elevated (0.63 vs. 0.28, *P* = 0.20). Therefore, we examined if other key glycolytic enzymes are elevated in burn skin in an analogous fashion to keloids.

We demonstrated a similar trend in burn skin, and markers of glucose uptake including *GLUT1* and *GLUT3* were increased compared with normal skin (1.24 vs. 0.27, *P* < 0.05, for *GLUT1*; 2.48 vs. 0.81, *P* < 0.05, for *GLUT3*). Furthermore, *HK1*, *HK2*, *PFK1*, *PDK1*, and *PKM2* expression was elevated compared with normal skin (2.84 vs. 0.81, *P* < 0.05, for *HK1*; 2.36 vs. 0.32, *P* < 0.01, for *HK2*; 3.83 vs. 0.95, *P* < 0.05, for *PFK1*; 4.31 vs. 0.76, *P* < 0.0001, for *PDK1*; 2.98 vs. 0.28, *P* < 0.01, for *PKM2*) (Figure 3B, center panel). Similarly, we demonstrated increased protein expression for Glut1 and PKM2 in burn skin compared with normal skin (1.03 vs. 0.31, *P* < 0.05, for Glut1, 1.48 vs. 0.63, *P* = 0.07, for PKM2) (Figure 3, C and D).

Although glycolytic alterations in burn skin mimicked those seen in keloids, upregulation of glycolysis occurs in a spectrum between physiologic burn wound healing and scarring (Figure 3B, right). To complement the results from gene and protein analysis, functional studies using a Seahorse XF96 glycolysis stress test were performed on fibroblasts from normal skin, burn, and keloid tissues (Figure 3E). Indeed, in comparison with normal skin, glycolysis (Figure 3F) and glycolytic capacity (Figure 3G) are markedly increased in the burn and keloid groups, thus highlighting the importance of glucose metabolism in these pathological states. In order to regulate burn skin glycolysis, we subsequently targeted PKM2, the final rate-limiting step in the glycolytic pathway.

### Targeting PKM2-mediated glycolysis decreases lactate production and inflammation in human burn skin.

**Figure 4:**
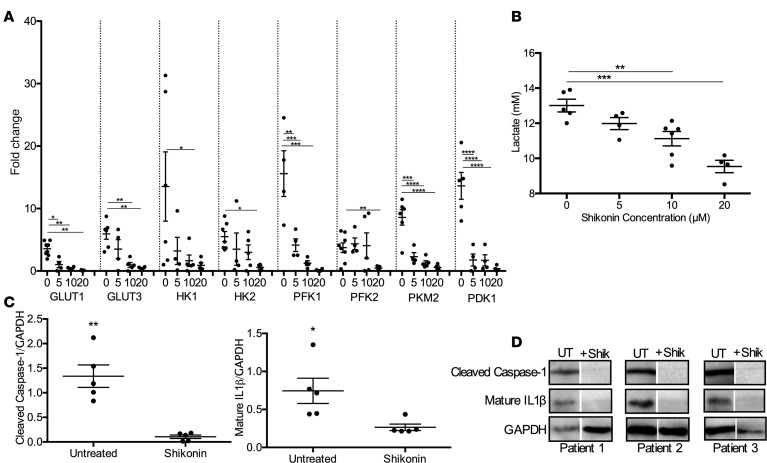
Shikonin treatment diminishes expression of human glycolytic enzymes and inflammatory by-products. (**A**) Gene expression of GLUT1, GLUT3, HK1, HK2, PFK1, PFK2, PKM2, and PDK1 after a 48-hour treatment with 0, 5, 10, or 20 μM shikonin (*n* = 4–5). (**B**) Secreted lactate levels in media collected from untreated and shikonin-treated burn fibroblasts (*n* = 4–6). (**C**) Protein expression of cleaved caspase-1 (*n* = 5) and mature IL-1β (*n* = 5) in untreated and shikonin-treated skin (20 μM). (**D**) Representative cropped Western blots for cleaved caspase-1 and mature IL-1β. Values are expressed as average fold change relative to normal skin, presented as mean ± SEM. Experiments were conducted twice. Student’s *t* test and 1-way ANOVA; *P < 0.05, **P < 0.01, ***P < 0.001, and ****P < 0.0001.

Inhibition or knockdown of PKM2 was shown to impair inflammation and lactate production in vitro, which are components of normal postburn wound healing and are critical prerequisites to keloid formation (23). To determine if blocking PKM2 can downregulate key glycolytic genes in burn skin, we successively treated burn skin (0–3 days after burn, average age 40 years and TBSA 15%) with a potent PKM2 inhibitor, shikonin (Figure 4A). Treatment with 5–10 μM of the glycolysis inhibitor shikonin significantly decreased gene expression of the facilitative glucose transporters, *GLUT1* and *GLUT3* (3.64 vs. 1.02, *P* < 0.05 with 5 μM for *GLUT1*; 5.94 vs. 0.98, *P* < 0.01 with 10 μM for *GLUT3*). Furthermore, shikonin downregulated expression of the rate-limiting enzymes *HK1* and *HK2*, although a 20 μM dose was needed (13.0 vs. 0.91, *P* < 0.05, for *HK1*; 5.50 vs. 0.55, *P* < 0.05, for *HK2*). Shikonin treatment also downregulated the rate-limiting enzyme *PFK1* (15.6 vs. 4.13, *P* < 0.01) with all dosages, while *PFK2* expression was only significantly lowered with a 20 μM dose (3.73 vs. 0.46, *P* < 0.01). Finally, we demonstrated here that 5 μM shikonin treatment inhibits gene expression of *PKM2* (8.59 vs. 2.30, *P* < 0.001) and downregulated *PDK1* (13.6 vs. 1.76, *P* < 0.001), which is in line with previous work (23). This was coupled with decreased lactate production at higher concentrations of shikonin (10 μM and 20 μM) (Figure 4B). Taken together, these results suggest that administration of shikonin effectively downregulated key enzymes involved in glucose uptake, glycolysis, and lactate production, all of which are important features of fibrotic and hyperproliferative conditions.

Interestingly, shikonin treatment also targeted NLRP3-mediated inflammation. Treatment of burn skin with 20 μM shikonin in vitro decreased protein levels of cleaved caspase-1 (0.30 vs. 0.09, *P* < 0.05) and mature IL-1β (3.61 vs. 0.26, *P* < 0.01) (Figure 4, C and D). Although sustained activation of inflammatory and glycolytic pathways are integral features of aberrant wound healing and scarring, NLRP3-mediated inflammation and aerobic glycolysis are also key components of normal wound healing. Therefore, we next assessed the effect of shikonin on burn wound healing in vivo using a murine model.

### PKM2 inhibitor shikonin is beneficial for wound healing in mice.

**Figure 5:**
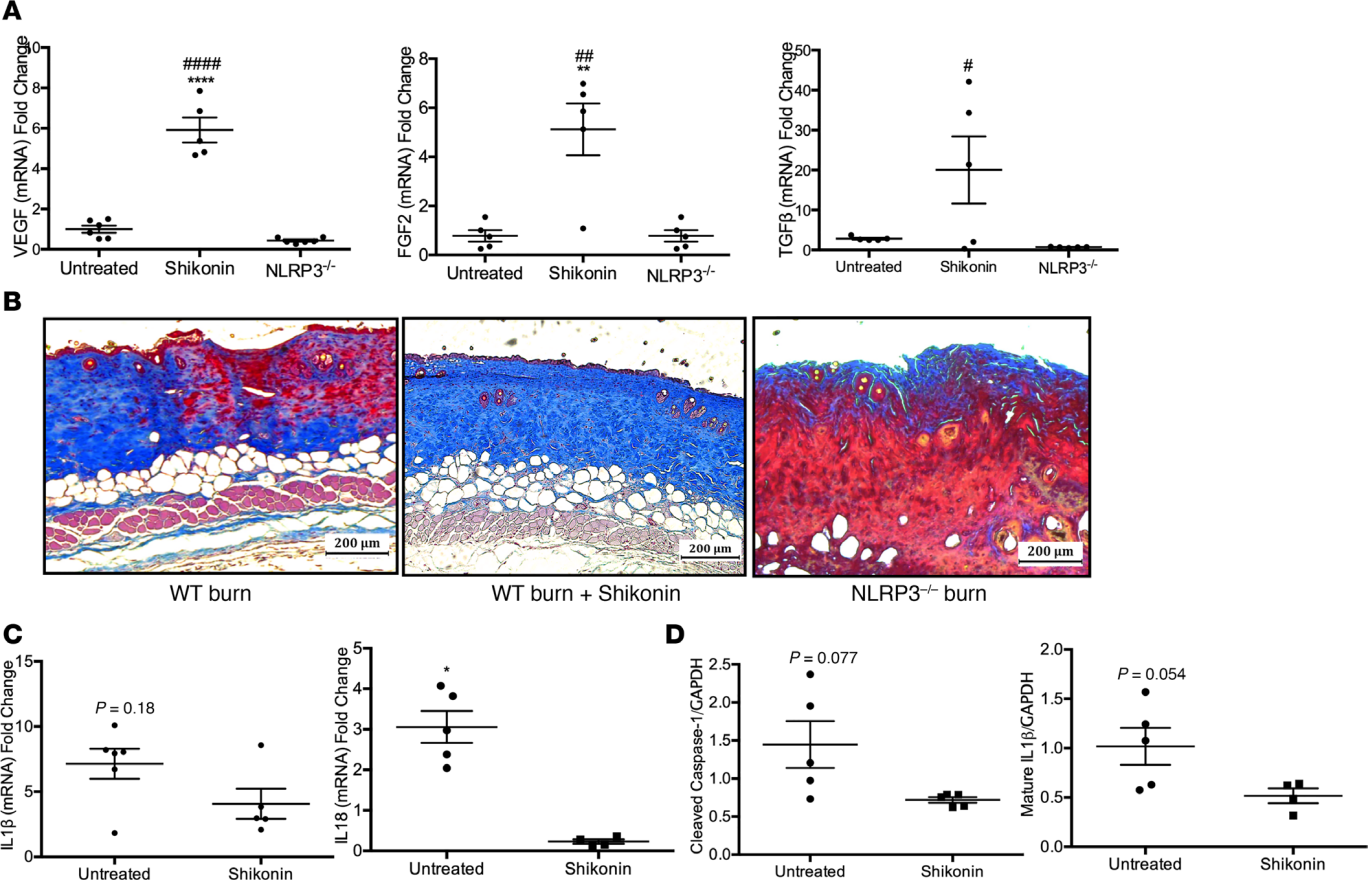
Shikonin treatment is not detrimental to wound healing in vivo. (**A**) Gene expression of *VEGF*, *FGF2*, and *TGFβ* at 7 days after burn (*n* = 5–6). (**B**) Trichrome staining of excised burn skin from untreated WT burn (left), treated WT burn (center), and untreated NLRP3^–/–^ burn (right) mice indicates increased dermal collagen deposition–treated mice. (**C**) Murine skin gene expression for *IL1β* (*n* = 5–6) and *IL18* (*n* = 4–6) at 7 days after burn. (**D**) Protein expression for cleaved caspase-1 and mature IL-1β in untreated and shikonin-treated murine skin (*n* = 4–5). Values are expressed as average fold change relative to normal skin, presented as mean ± SEM. Experiments were conducted twice. One-way ANOVA with Tukey’s post hoc or Students *t* test; *P < 0.05 and **P < 0.01; ***P < 0.001; ****P < 0.0001 shikonin versus untreated; ^#^*P* < 0.05, ^##^*P* < 0.01, ^###^*P* < 0.001, and ^####^*P* < 0.0001 shikonin versus NLRP3^–/–^.

We administered shikonin in mice and compared levels of the growth factors *VEGF*, *FGF2*, and *TGFβ* between untreated, shikonin treated, and NLRP3^–/–^ burn skin (Supplemental Figure 3; supplemental material available online with this article; https://doi.org/10.1172/jci.insight.138949DS1). Treated mice demonstrated significantly increased gene expression of *VEGF*, *FGF2*, and *TGFβ* compared with untreated and NLRP3^–/–^ mice (5.91 vs. 1.00, *P* < 0.001, and 5.91 vs. 0.44, *P* < 0.001, for *VEGF*; 5.12 vs. 0.71, *P* < 0.01, and 5.12 vs. 0.78, *P* < 0.01, for *FGF2*; 20.1 vs. 2.84, *P* < 0.05, and 16.7 vs. 0.72, *P* < 0.05, for *TGFβ*) at 7 days after burn (Figure 5A). Subsequently, we performed trichrome staining to assess wound healing in shikonin-treated mice (Figure 5B). Trichrome staining demonstrated increased dermal collagen deposition and keratinization compared with untreated and NLRP3^–/–^ mice, confirming that shikonin treatment does not impair burn wound healing. Likely, this is due to the fact that this and lower doses of shikonin inhibit glycolysis but simply attenuate NLRP3 activation in vivo. Indeed, we showed decreased gene expression for *IL1β* (7.0 vs. 4.1, *P* = 0.18) and *IL18* (3.1 vs. 0.23, *P* < 0.05) with shikonin, which was only significant for IL-18 (Figure 5C). Similarly, while cleaved caspase-1 and IL-1β cleavage decreased with shikonin administration, there was no significant difference between untreated and treated burn skin (1.45 vs. 0.72, *P* = 0.077, for cleaved caspase-1; 1.02 vs. 0.52, *P* = 0.054, for mature IL-1β) (Figure 5D).

While shikonin did not interfere with normal burn wound healing in mice, identifying and selectively treating patients who are at risk for abnormal wound healing is key. Identifying this subset of patients based on local glucose uptake and metabolism (e.g., Glut1 expression) would allow for personalized treatment and would minimize poor wound healing outcomes in normal burn patients. While further work is needed at this point, targeting both NLRP3-mediated inflammation and aerobic glycolysis could be an effective strategy in these patients.

## Discussion

Effective wound healing after burns is a critical predictor of patient outcomes. While impaired healing is a concern in deep dermal or full-thickness burns, a subset of these patients may suffer from aberrant wound healing such as posttrauma scarring. Excessive scarring can occur in the form of keloids, which recapitulate the major clinical features associated with benign skin tumors, including uncontrolled growth, invasion of normal tissues, and recurrence despite treatment (2, 3). In addition to gross and histological similarities, keloids exhibit metabolic and inflammatory alterations that parallel those seen in tumors such as chronic inflammation and an unstimulated increase in glucose uptake (25, 26). Owing to its tumor-like dependence on glucose, we hypothesized that development of keloids is predicated on greater posttrauma glucose availability, uptake, and upregulation of key glycolytic enzymes in burn skin. Therefore, burn patients who eventually develop keloids potentially show early evidence of the aforementioned features compared with nonkeloid patients.

In this study, we investigated (a) if NLRP3-mediated inflammation is activated in keloids, (b) whether Glut1 expression is elevated in burn tissue from keloid compared with nonkeloid patients, (c) if glycolytic enzymes are upregulated in burn skin in a similar fashion to keloids, and (d) if the PKM2 inhibitor shikonin downregulates inflammatory markers and glycolytic enzymes in burn skin while sparing normal wound healing. We showed evidence of NLRP3 inflammasome activation and overexpression of Glut1 in keloid tissue compared with burn and normal skin. Intriguingly, there was a difference between burn skin from keloid and nonkeloid patients with regard to Glut1 expression. Skin obtained from the former exhibited enhanced basal epidermal and dermal Glut1 staining and gene expression. Therefore, we proposed that significantly elevated Glut1 expression and, hence, glucose uptake is an indicator for increased keloid risk after burns.

Although Glut1 is critical for glucose uptake and, subsequently, aerobic glycolysis, upregulation of glycolytic enzymes has a critical role, as well. Fibrotic conditions demonstrate activation of glycolysis via upregulation of key glycolytic enzymes, including HK1/2, PFK1/2, and PKM2 (4). Since hyperproliferative conditions depend on glycolysis, drugs targeting these glycolytic enzymes in burn skin could be an attractive preventative strategy (27, 28). Ideally, more efficient downregulation of glycolysis could be achieved via inhibitors such as shikonin, which suppress multiple steps in the glycolytic pathway and could serve as a powerful means to mitigate abnormal wound healing responses after burn. Interestingly, while shikonin is known to target key glycolytic enzymes, we showed here that it downregulates inflammatory pathways, as well (29). Since inflammation and aerobic glycolysis are integral to normal wound healing, using a lower dose or limiting therapy to high-risk patients would mitigate the possibility of impaired wound healing in normal patients (low risk of abnormal scarring) (30, 31).

While the data presented suggest that targeting glycolysis and NLRP3-mediated inflammation is a possible therapeutic strategy, there are several limitations. First, comparison of patient characteristics indicated that average age of the keloid burn patients was lower than nonkeloid counterparts (Supplemental Figure 1). However, there was no significant difference with regard to injury characteristics, and patients were matched based on TBSA and burn etiology. Another study limitation was that we discussed inflammation solely in the context of NLRP3 activation. Although we focused on NLRP3 since it is activated in burn tissue and metabolic disease states (e.g., diabetes and obesity), we cannot exclude the fact that other NLRs could be involved and could compensate for inhibition of NLRP3 activation. However, recent studies underscore the link between NLRP3-mediated inflammation and cellular metabolism — a metabolically triggered inflammatory state known as “metainflammation,” which is a form of chronic, low-grade inflammation accompanying metabolic disorders (32). Therefore, NLRP3 may have a more important function in local skin metabolic derangements after burns as opposed to other NLRs. Furthermore, we proposed that dysregulated glycolysis could promote a chronic inflammatory state based upon previous studies demonstrating that PKM2-mediated glycolysis enhances NLRP3 activation via lactate-mediated phosphorylation of EIF2AK2 (33). However, it is important to note that further work is needed to completely elucidate the link between NLRP3-mediated inflammation and glycolytic alterations in keloids. Additionally, we proposed that Glut1 is a potential indicator for abnormal glucose metabolism and increased keloid risk. However, there are limitations with regard to the positive predictive value of 1 marker and, ideally, a gene-screening panel that includes other glycolytic enzymes would be of merit. While identifying patients with evidence of aberrant glucose metabolism and treatment with glycolysis inhibitors could be promising, further studies are needed at this point.

## Methods

### Patients.

Patients admitted to the Ross Tilley Burn Center at Sunnybrook Hospital (Toronto, Canada) were consented preoperatively for tissue collection. We enrolled 42 patients with a minimum TBSA of 20% (Supplemental Figure 1). Of the 42 patients, 32 patients (23 males, 9 females) with 38.9% ± 3.8% TBSA burns were allocated to the nonkeloid burn group. The remaining 10 burn patients (6 males, 4 females) who developed keloids within 2 years after injury (average time to presentation 458 ± 98 days) with 43.4% ± 11.4% TBSA burns were assigned to the keloid burn group. From this group, we obtained burn skin samples during admission and keloid tissue on follow-up. Keloid tissue was identified based on clinical features (e.g., location, appearance, extension beyond wound margins) (Supplemental Figure 2) (34). For controls, we obtained skin from 5 nonburn patients (normal) undergoing elective surgery. Skin was obtained at various time points after burn (1 specimen per patient) and grouped into postburn day ranges in order to increase sample size (Table 1 and Table 2). Day ranges were selected based on previous work (15).

### Animals and model.

WT C57BL/6J (WT) and NLRP3-KO (NLRP3^–/–^) male mice (6–8 weeks old) were purchased from Jackson Laboratory, housed at ambient temperature, and cared for in accordance with the *Guide for the Care and Use of Laboratory Animals* (National Academies Press, 2011). All mice were anesthetized with 2.5% isoflurane and shaved along the dorsal spine region. Ringers lactate (2–3 mL) was injected s.c. in all treatment mice to protect the spine, and buprenorphine (0.05–0.1 mg/kg body weight) was injected for pain management. A full-thickness, third-degree scald burn encompassing 30%–35% TBSA was induced by immersing the dorsal region in 98°C water for 10 seconds and the ventral region for 2 seconds. Sham mice (control) underwent identical procedures except the burn. Mice were sacrificed at 7 days after burn.

### Histology and IHC.

Skin was collected and immediately fixed in 10% formalin and then maintained in 70% ethanol before paraffin embedding. Subsequently, tissues were sectioned and incubated with Glut1 antibody (Abcam, 115730) or NLRP3 antibody (Abcam, 214185), followed by DAB staining. For Masson’s trichrome staining, paraffin-embedded slides were heated for 30 minutes at 60°C. The slides were then deparaffinized with citrosol, followed by rehydration through 100% twice, 95%, 70%, and washed in distilled water. Slides were placed in Bouin’s solution (26367–01; EMS) for 1 hour at 56°C and washed. Hematoxylin stain (HHS16; MilliporeSigma) and Biebrich scarlet-acid fuchsin solution were applied sequentially for 10 minutes. After each stain, the slides were washed. Next, slides were differentiated in phosphomolybdic–phosphotungstic acid for 15 minutes and transferred to aniline blue for 5 minutes. All slides were washed in distilled water and then differentiated in 1% acetic acid for 2 minutes. Slides were dehydrated through 95% ethanol and absolute ethanol, followed by clearing in citrosol, and were mounted with SHUR/Mount xylene-based liquid mounting media (Triangle Biomedical Sciences). Imaging was performed on an LSM confocal microscope.

### Shikonin treatment.

Human burn skin was obtained postoperatively and was immediately transferred to the laboratory for preparation. Tissue was cut into small pieces, and 10 grams of diced burn skin was incubated in a culture plate (Thermo Fisher Scientific) with 50 mL of DMEM (low glucose; Wisent) supplemented with 10% FBS (Wisent) and 1% penicillin/streptomycin (Thermo Fisher Scientific) in an atmosphere of 5% CO_2_ at 37°C. Media was supplemented with 0, 5, 10, or 20 μM shikonin (Cayman Chemical, 517-89-5) and was replaced after 24 hours. Treated skin was collected at 48 hours. For fibroblast cultures, tissue from 6 different burn patients and 5 keloid patients (randomly selected from enrolled patients) was cut into small pieces and digested with collagenase (Invitrogen), dispase II (Roche), and 0.05% trypsin (Thermo Fisher Scientific) at 37°C for 1 hour. The enzymes were neutralized by adding DMEM with 10% FBS and 1% penicillin/streptomycin, after which the suspension was centrifuged (383*g*, 37°C, 5 minutes). The cell pellet was resuspended in DMEM and passed through a 40-μM cell strainer. Cell viability was assessed using Trypan blue, and a total of 20,000 cells/well was resuspended in 2 mL of DMEM and plated in 6-well plates. Media supplemented with 0, 5, 10, or 20 μM shikonin was added to each well when the cells reached 90% confluence. Media was removed and replaced with fresh DMEM for another 24 hours after the wash out period (first 24 hours). For animal experiments, WT mice were injected i.p. daily with shikonin (10 mg/kg) for 7 days. This dose was chosen based on previous studies demonstrating efficacy (35).

### Lactate levels.

Secreted lactate levels were measured from media obtained from untreated and shikonin-treated cultured burn tissue. Lactate was measured using an L-lactate assay kit according to the manufacturer’s instructions (Abcam, ab65331).

### Gene expression using RT-PCR.

Total RNA isolated from skin tissue was analyzed by quantitative PCR (qPCR). RNA was isolated from tissue and cells using TRIzol-chloroform (Invitrogen) with subsequent purification using the RNeasy Kit (QIAGEN) according to the manufacturer’s instructions. RNA (2 μg) was transcribed to cDNA using the high-capacity cDNA reverse transcription kit (Applied Biosystems). qPCR was performed using the Applied Biosystems Step One Plus Real-Time PCR System. Primer sequences used are as follows (listed as forward and reverse, respectively): *GLUT1* (5′-TCAACACGGCCTTCACTG-3′ and 5′-CACGATGCTCAGATAGGACATC-3′), *GLUT3* (5′-GACCCAGAGATGCTGTAATGGT-3′ and 5′-GGGGTGACCTTCTGTGTCCC-3′), *HK1* (5′-GGTGAAATCGTCCGCAAC-3′ and 5′-CCGGGTCTTCATCGTC-3′), *HK2* (5′-ATTGTCCAGTGCATCGCGGA-3′ and 5′-AGGTCAAACTCCTCTCGCCG-3′), *PFK1* (5′-CGGAAGTTCCTGGAGCACCTCTC-3′ and 5′-AAGTACACCTTGGCCCCCACGTA-3′), *PFK2* (5′-CCTCGTTGCCCAGATCCTGT-3′ and 5′-GCTAAGGCACATTGCTTCCG-3′), PDK1 (5′-TCCCCCGATTCAGGTTCAC-3′ and 5′-GTGAGCACTCCTGCCAGACT-3′), *PKM2* (5′-CCACTTGCAGCTATTCGAGGAA-3′ and 5′-GTGAGCACTCCTGCCAGACT-3′), *VEGF* (5′-GGAGACTCTTCGAGGAGCACTT-3′ and 5′-GGCGATTTAGCAGCAGATATAAGAA-3′), *FGF2* (5′-GTGTGTFCCAACCGGTACCT-3′ and 5′-GCTCTTAGCAGACATTGGAAG-3′), and *TGFβ* (5′-TGACGTCACTGGAGTTGTACGG-3′ and 5′-GGTTCATGTCATGGATGGTGC-3′). Gene expression was expressed relative to β-actin.

### Western blotting.

Protein from human tissue, keloid tissue, and murine skin was extracted in RIPA buffer containing phosphatases and proteases inhibitor cocktails (Roche). Protein concentrations were determined by the BCA protein assay kit (Pierce). Proteins were resolved by SDS-PAGE followed by Western blotting using the following antibodies at 1:1000 concentration: cleaved caspase-1 (Cell Signaling Technology, D57A2), cleaved IL-1β (Cell Signaling Technology, D3A3Z), Glut1 (Abcam, ab15309), PKM2 (Cell Signaling Technology, 4053), Hif1α (Cell Signaling Technology, 36169), and GAPDH (Cell Signaling Technology, 5174). Species appropriate secondary antibodies conjugated to horseradish peroxidase (Bio-Rad) were used, and proteins were visualized by enhanced chemiluminescence using the Bio-Rad ChemiDoc MP Imaging System. Band intensities were detected, normalized, and quantified with the ChemiDoc and Image Lab 5.0 software (Bio-Rad). Western blot samples for identical markers and species were derived from the same experiment and were processed in parallel. Antibody concentrations are expressed relative to GAPDH.

### Seahorse XF96 glycolysis stress test.

Fibroblasts were seeded at a density of 3 × 10^4^ cells/well as per the Seahorse protocol (Agilent). Once cells had adhered, the standard medium was washed out and replaced with XF Base Medium (+ 2 mM L-glutamine); then, the plate was incubated at 37°C without CO_2_ for 45 minutes. Once loaded into the Seahorse analyzer, extracellular acidification rate (ECAR) was measured at baseline as well as after injection of D-glucose (10 mM), oligomycin (1 μM), and 2-deoxy-D-glucose (50 mM). The measured data were subsequently analyzed using the XF glycolysis test report generator.

### Statistics.

All data are represented as mean ± SEM. Statistical analysis was performed using 2-tailed Student’s *t* test, χ^2^ test, 1- and 2-way ANOVA, and Mann-Whitney *U* test to compare groups, where appropriate. All graphs were created using GraphPad Prism 6.0 and analyzed statistically using SPSS 20 (IBM), with significance accepted at *P* < 0.05.

### Study Approval.

Animal experiments were conducted in accordance and approved by the Sunnybrook Research Institute Animal Care Committee (Toronto, Ontario, Canada), protocol no. 467. For human studies, approval was obtained from the Research Ethics Board at Sunnybrook Hospital (REB 194-2010). Written informed consent was received from participants before study inclusion. Mice were cared for in accordance with the *Guide for the Care and Use of Laboratory Animals* (National Academies Press, 2011).

## Author contributions

RV designed research studies, performed experiments, acquired and analyzed data, and wrote portions of the manuscript; DB analyzed data and wrote portions of the manuscript; CA performed experiments, analyzed data, and wrote portions of the manuscript; AA edited and wrote portions of the manuscript; and MJ designed research studies and wrote portions of the manuscript.

## Supplementary Material

Supplemental data

## Figures and Tables

**Table 2:**
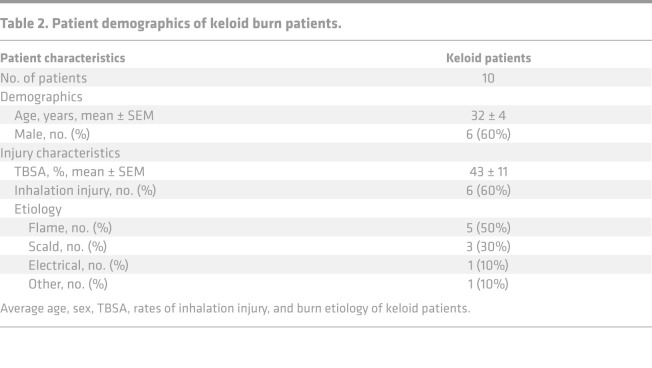
Patient demographics of keloid burn patients.

**Table 1:**
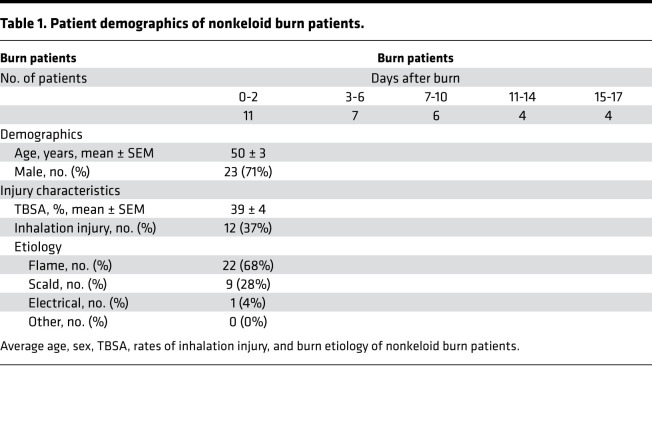
Patient demographics of nonkeloid burn patients.

## Version 1 08/04/2020

In-Press Preview

## Version 2 09/03/2020

Electronic publication
