# Supplementary material for: Regulation of glycolysis and the Warburg effect in wound healing
Source: JCI Insight. 2020 Sep 3;5(17):e138949. doi: 10.1172/jci.insight.138949 (PMC7526439; doi:10.1172/jci.insight.138949)
Supplement: Supplemental data [file jciinsight-5-138949-s260.pdf]

Patient Characteristics	Burn Patients	Keloid Patients	P*
No. of Patients	32	10	
Demographics			
Age, years, mean ± SEM	50 ± 3	32 ± 4	**0.0017***
Male, no. (%)	23 (71%)	6 (60%)	0.4783
Injury characteristics			
TBSA, %, mean ± SEM	39 ± 4	43 ± 11	0.7151
Inhalation injury, no. (%)	12 (37%)	6 (60%)	0.1844
Etiology			
Flame, no. (%)	22 (68%)	5 (50%)	0.2801
Scald, no. (%)	9 (28%)	3 (30%)	0.9088
Electrical, no. (%)	1 (4%)	1 (10%)	0.3729
Other, no. (%)	0 (0%)	1 (10%)	0.2381

*significant differences between burn and keloid groups ($p<0.05$)

Figure S1 Statistical comparison of burn and keloid patient characteristics.

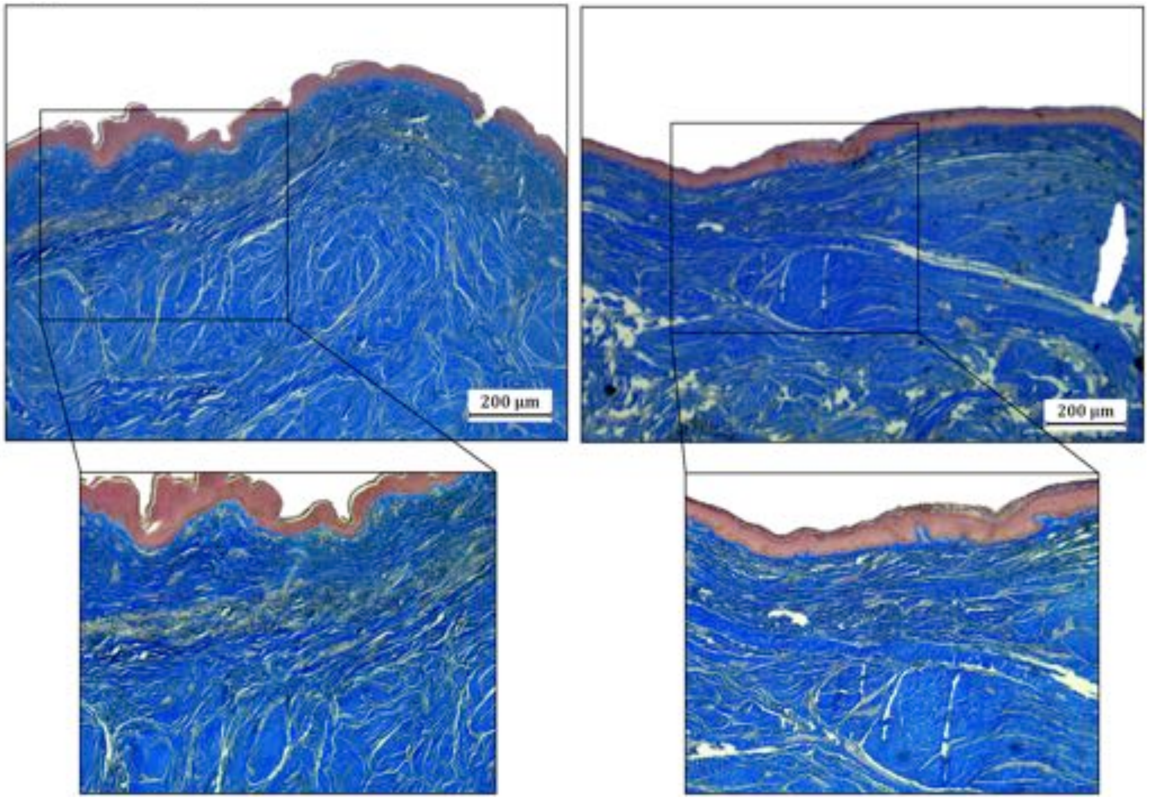

Figure S2 Trichrome staining in post-burn keloid tissue indicates thicker, disorganized collagen fibers.

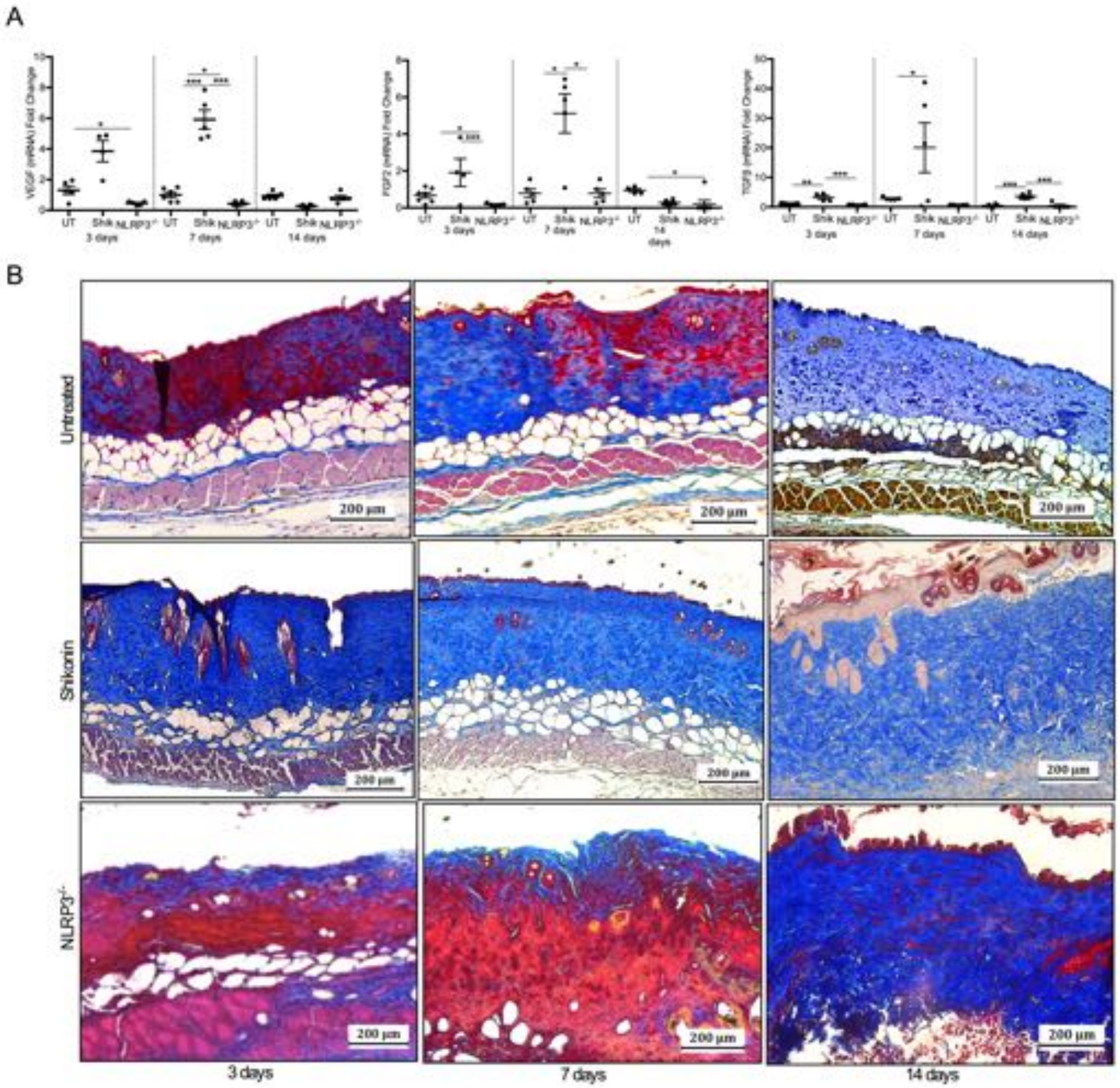

Figure S3 Growth factor gene expression and trichrome staining in murine skin for shikonin versus untreated and NLRP3$^{-/-}$.
